# Diseases of the Nervous System

**Published:** 1892-08

**Authors:** 


DISEASES OF THE NERVOUS SYSTEM.—By J. A. Ormerod, M. D., Oxon., F. R. C. P., London, Medical Registrar and Demonstrator of Morbid Anatomy at St. Bartholomew's Hospital; Physician to the National Hospital of the Paralyzed and Epileptic, Queen Square, and to the City of London Hospital for Diseases of the Chest, Victoria Park. Published by P. Blakiston, Son & Co., Philadelphia, Pa.

THIS work is good. Professor Ormerod has succeeded in condensing a remarkable amount of thought and knowledge in a very few pages. This work, like most condensed essays, fails at times to give as elaborate description as might be desired; but space oftentimes compels an author to abbreviate description, when he might otherwise do himself greater justice and his readers good.

We have seen no work on the subject which gives more substance in the same space. His figures and diagrams are well wrought and instructive, and his anatomy up to the latest investigations.

